# Designing, development and validation of an app to reduce the response time of the emergency medical services

**DOI:** 10.1371/journal.pone.0299828

**Published:** 2024-03-25

**Authors:** Júlia Loverde Gabella, Iago Amado Peres Gualda, Isadora Laguila Altoé, Matheus Henrique Arruda Beltrame, Pedro Henrique Aguillar da Silva, Dalton Breno Costa, Fernando César Grossi Paggi, Sérgio Sanches Fabres Filho, Miyoko Massago, Luiz Gustavo de Paulo, Marcos Rogério Bitencourt, Anjni Patel Joiner, João Ricardo Nickenig Vissoci, Luciano de Andrade

**Affiliations:** 1 Department of Medicine, State University of Maringá, Maringá, Paraná, Brazil; 2 Center for Global Emergency Medicine Innovation and Implementation, Duke Global Health Institute, Duke University, Durham, North Carolina, United States of America; 3 Department of Engineering, Unicesumar, Maringá, Paraná, Brazil; 4 Postgraduate Program in Health Sciences, State University of Maringá, Maringá, Paraná, Brazil; 5 Postgraduate Program in Management, Technology and Innovation in Urgency and Emergency, Department of Medicine, State University of Maringá, Maringá, Paraná, Brazil; 6 Department of Emergency Medicine, Duke University School of Medicine, Durham, North Carolina, United States of America; 7 Duke Global Health Institute, Duke University, Durham, North Carolina, United States of America; FIOCRUZ: Fundacao Oswaldo Cruz, BRAZIL

## Abstract

**Introduction:**

Delays in prehospital care attributable to the call-taking process can often be traced back to miscommunication, including uncertainty around the call location. Geolocation applications have the potential to streamline the call-taking process by accurately identifying the caller’s location.

**Objective:**

To develop and validate an application to geolocate emergency calls and compare the response time of calls made via the application with those of conventional calls made to the Brazilian Medical Emergency System (*Serviço de Atendimento Médico de Urgência*—SAMU).

**Methods:**

This study was conducted in two stages. First, a geolocating application for SAMU emergency calls (CHAMU192) was developed using a mixed methods approach based on design thinking and subsequently validated using the System Usability Scale (SUS). In the second stage, sending time of the geolocation information of the app was compared with the time taken to process information through conventional calls. For this, a hypothetical case control study was conducted with SAMU in the Maringá, Paraná, Brazil. A control group of 350 audio recordings of emergency calls from 2019 was compared to a set of test calls made through the CHAMU192 app. The CHAMU192 group consisted of 201 test calls in Maringá. In test calls, the location was obtained by GPS and sent to the SAMU communication system. Comparative analysis between groups was performed using the Mann-Whitney test.

**Results:**

CHAMU192 had a SUS score of 90, corresponding to a “best imaginable” usability rating. The control group had a median response time of 35.67 seconds (26.00–48.12). The response time of the CHAMU192 group was 0.20 (0.15–0.24).

**Conclusion:**

The use of the CHAMU192 app by emergency medical services could significantly reduce response time. The results demonstrate the potential of app improving the quality and patient outcomes related to the prehospital emergency care services.

## 1 Introduction

Rapid access to care for time-dependent emergencies is crucial for improving outcomes. Each segment of the chain of survival can be optimized to improve overall care and time to in-hospital treatment. Prehospital emergency care (PEC) comprises many key segments of the chain of survival, including early activation of the emergency response system through notification of the emergency call center, on-scene care and subsequent transfer to in-hospital teams [1]. Digital technologies can accurately locate callers and patients and have the potential to improve emergency personnel response, thereby reducing overall PEC response time [2, 3].

Activation of the PEC system traditionally involves either a patient or bystander using a phone to call the closest emergency call center. The call is then typically processed by a telecommunicator through targeted questions, subsequently resulting in a triaged response by prehospital personnel. Although this conventional PEC call process is widely used throughout the world, many of its aspects remain under discussion, particularly with regarding to call-processing times (CPT), which comprises from the initial call to the emergency center to the time of ambulance dispatch [4]. CPT can be impacted by a multitude of factors, including language barriers, location uncertainty, and confusion or disorientation of the caller [5]. Furthermore, if the caller is unable to relay an accurate address of the emergency location, this can result in further delays to treatment and subsequent transport.

The widespread availability of cellular phones and WiFi networks provides a unique opportunity to leverage these technologies in emergency call scenarios. The Global Positioning System (GPS) can be used to geolocate callers and accurately identify the location of the emergency within 10 m [2]. Prior pilots evaluating the feasibility of using automatic geolocation within cellular phone applications for emergency calls have demonstrated both accuracy and reduced times to initial treatment [3].

This study aimed to evaluate the functional and technical performance of mobile phone application prototype designed to accurately locate the user and activate the PEC system to reduce overall response time in a medium-large city in southern Brazil. The application prototype was developed using design thinking and the Flutter. Validation was performed in two steps: technical evaluation by health professionals and comparison of the application with conventional call times for sending geolocation information.

## 2 Materials and methods

This study refers to the development of a technological instrument using the Design Thinking methodology which consists of five stages: namely discovery, definition, development, delivery, and validation [6].

### 2.1 Activation of the EMS response in Brazil

*Serviço de Atendimento Médico de Urgência* (SAMU) is the national PEC system in Brazil [7]. The PEC response in Brazil is triggered by a toll-free call to number 192. The call is subdivided into temporal strata, each of which represents a layers or divisions of time from reception, evaluation, and triaging for user’s calls. Calls are then prioritized based on severity, and appropriate resources are promptly dispatched. By analyzing additional time intervals within the activation of EMS response time, it is possible to better understand the processes related to assessing EMS performance.

**Fig 1:**
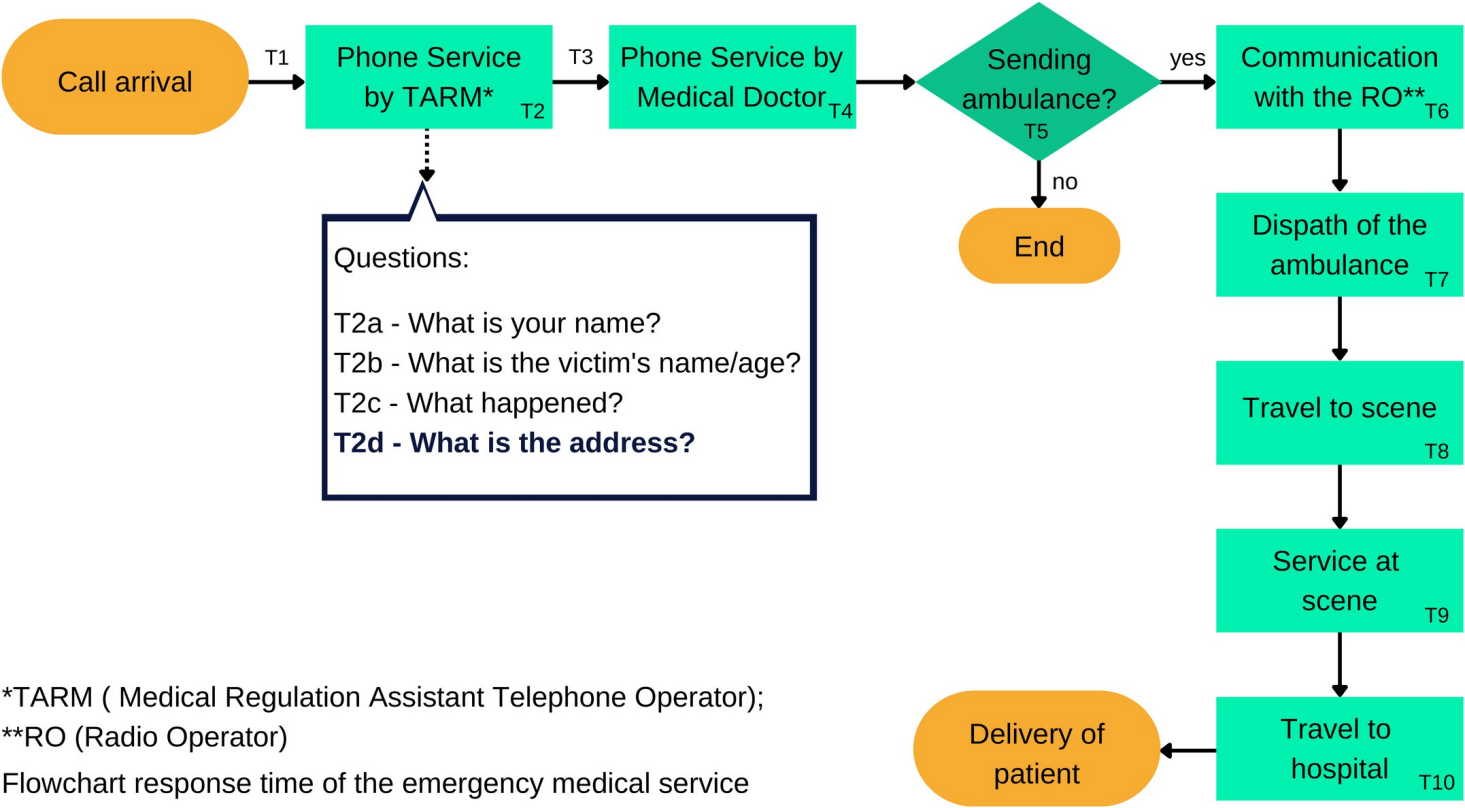
Flowchart of time segments of the emergency medical service responses.

PEC responses in Brazil can be divided into 10 separate time intervals, as shown in the flowchart of Fig 1. There are small variations of this flowchart according to adaptations made in each EMS unit [7]. The total CPT is the sum of strata T2, T3 and T4. Current recommendations are that the total CPT should not exceed 3 min [8]. EMS activation time (T2) can range from 30 s to 1 min. T2 corresponds to the time that the Medical Regulation Assistant Telephone Operator (MRATO) communicates with the caller. MRATO is the Assistant Telephone Operator who first talks to the victim and then passes the case to the Medical Doctor. The MRATO makes the following questions to callers: (T2a)–“What is your name?”; (T2b)—“What is the victim’s name and age?”; (T2c)—“What happened?” and (T2d)—“What is the scene address?” [8, 9]. In our paper, the question T2d is the central point of the time analysis.

### 2.2 Ethics committee

This study was approved by the Standing Committee on Human Research Ethics in Research at State University of Maringá, pursuant to CAAE 47967621.2.0000.0104. The study was authorized by Maringá City Hall, SAMU Regional Norte Novo, and the Standing Committee on Ethics of the State University of Maringá. The audio sample of emergency calls was obtained retrospectively from the occurrence registration system of SAMU Regional Norte Novo, ensuring anonymity and protection of personal information. Analysis of SAMU audio recordings was authorized by the ethics committee. At all other stages of the research, the participants were informed about the risks and benefits of participation. Volunteers individually confirmed their participation and authorized the use of information by completing an online informed consent form, following the parameters previously approved by the ethics committee. Individuals under the age of 18 years were not included in the study. For compliance with data confidentiality and reliability legislation, the information generated by volunteers was stored and treated in accordance with Law No. 13.709/2018 (Brazilian General Data Protection Law) [10].

### 2.3 Inclusivity in global research

Additional information regarding the ethical, cultural, and scientific considerations specific to inclusivity in global research is included in the Supporting Information (S1 Checklist).

### 2.4 Data gathering requirements for app development

A mixed-methods approach was used to gain an understanding of both SAMU perspectives and the general public’s approach to communication during emergency situations. Through verified semi-structured interviews were conducted with call-takers (MRAT), the medical control physician and the dispatcher of SAMU Regional Norte Novo, Paraná state, (Brazil).

Interviews with the EMS team reveladed that pinpointing the full address of callers (street and house number) was a primary difficulty faced by call-takers, as their systems cannot work with an uncertain location or a general surrounding. On the basis of this information, we developed and applied a single-question survey to the general public, with the aim of assessing the behavior of the population in the face of an emergency. A convenience sample of participants was recruited via various social media networks (Instagram, Facebook and WhatsApp) [11–13]. Surveys conducted from October to December 2021.

Deidentified responses were collected using Google Forms platform [14]. The objective question was the following: *“Imagine you are in an urgent and/or emergency situation at an atypical geographic location*. *Would you know how to inform*, *via a phone call*, *in less than one minute*, *the complete address where you are located WITHOUT making use of geolocation tools*?*”*. The possible responses to the question were “YES” or “NO”. The question was presented in Brazilian Portuguese. A total of 266 completed.

### 2.5 App development

Development of the emergency call geolocation app was based on the Double Diamond design thinking method created by the British Design Council [6]. This approach was supported by a thorough literature search on the Pubmed database, perceptions of specialists and the general population, ideation of solutions and prototyping.

The app was designed using the free-access software Flutter for Android and iOS mobile application development [15]. Flutter was proposed by Google to encourage application development, natively compiled for mobile, web, and desktop environments. It is a rapid development framework written in Dart, that allows construction of screens in an expressive and flexible way through widget implementation.

In this project, the integrated development environment IDEA [16] was used. The environment compiles tools for software development, providing, in addition to the programming environment, simulators that allow a visualization of the developed interface. The database system used was MySQL [17], JPA [18] and Hibernate [19] to access the database. The stages of app development and validation are Results section.

### 2.6 Expert evaluation

Emergency medicine specialists were recruited to evaluate the developed app prototype. Participants were selected using a non-probabilistic, convenience sampling technique, excluding those who did not own an Android smartphone (version 7.1.2 Nougat onward). Given that there is no consensus on the ideal number of specialists, we chose to conduct interviews with eight experts [20].

Specialists signed informed consent form, were asked to complete a questionnaire created using Google Forms, and were sent a link to download the app via their email addresses. Participants accessed the user interface design, the trigger mechanism to send an emergency request with patient’s profile data, and the list of information required for pre-registration. The pre-registration information is sent to MRATO together with the geolocation coordinates. Questionnaire items were evaluated using the System Usability Scale (SUS) [21], without alterations or modification. Questionnaires were administered from January to July 2022.

The SUS scale is a quantitative analysis tool that consists of 10 items designed to measure the ease of use of a product in question (S1 Appendix). The respondents rated items on a 5-point Likert scale, ranging from 1 (“I strongly disagree”) to 5 (“I strongly agree”). Items were divided into two categories, namely positive and negative, as shown in Table 1 [21].

**Table 1:** Items of System Usability Scale [21] to access the adequacy of the proposed mobile application.

Positive items	Negative items
**(1)** I think that I would like to use this system frequently.	**(2)** I found the system unnecessarily complex.
**(3)** I thought the system was easy to use.	**(4)** I think that I would need the support of a technical person to be able to use this system.
**(5)** I found the various functions in this system were well integrated.	**(6)** I thought there was too much inconsistency in this system.
**(7)** I would imagine that most people would learn to use this system very quickly.	**(8)** I found the system very cumbersome to use.
**(9)** I felt very confident using the system.	**(10)** I needed to learn a lot of things before I could get going with this system.

The total score of experts was obtained by multiplying the sum of the individual responses by 2.5, resulting in a value between 0 and 100 [21]. The final score was classified into six categories. As follows: worst imaginable (≤ 20.5), poor (21.0–38.5), OK (39.0–52.5), good (53.0–73.5), excellent (74.0–85.5) and best imaginable (86.0–100.0) [22].

Agreement between experts’ responses was assessed using the Content Validity Coefficient (CVC), which is calculated in five steps [23]. In the first step, the mean value of experts’ scores (S) is calculated on the basis of SUS results. Next, the initial CVC (CVC_i_) of each item is estimated from the mean of scores (S) divided by the highest possible score of each item (in this case, 4).

In the third step, the polarization error (Pei) of experts is calculated to eliminate any biases. For this, the ratio of 1 to total number of specialists (k), raised to the k-th power is calculated. In the next step, the final CVC of each item (CVC_f_) is calculated by subtracting Pei from CVCi. Finally, the total CVC of the questionnaires (CVCt) is calculated as the difference between the mean of CVC_i_ and mean Pei [24].

### 2.7 Comparison of conventional call times and the app-mediated call times

The effectiveness of the app prototype was assessed by comparing the time of conventional calls and call taken trough the app (CAAE 47967621.2.0000.0104).

For conventional call data gathering, we used a convenience sample of audio recordings of emergency calls received by SAMU Regional Norte Novo, Paraná state, Brazil, from January to December of 2019. The data were accessed from January to July 2022. Of the total 182,273 calls received in 2019, only 5.49% were heard and individually analyzed using predefined categories of the Label Studio software. Of these, 2,326 were classified as urgent and emergency, and 350 were reproduced again to define the time spent by call-takers to gather information on the caller’s address (T2d) (control group). The authors had no access to information that could identify individuals during or after data collection.

For data collection from the app, the project team carried out the EMS activation test by performing test calls via the geolocation app at 201 different locations Maringá, Paraná, Brazil (CHAMU192 group) using Wi-Fi or mobile data (3G and/or 4G) from different telephone operators. Seeking to simulate the real distribution emergency situations, we carried out simulations in residential regions, public spaces and recreation areas [3]. The tests were conducted from August to September 2022.

Latency is an indicator of internet signal quality, representing the time it takes from a message to leave the source device, arrive at the destination, and return to the initial device [25]. Given that the caller does not need to verbalize their location to call-takers when using the app, the latency time of the app’s geolocation output to its visualization by the EMS call center was used as an equivalent variable to the T2d of conventional call. The geolocation of test calls via CHAMU192 was mapped using OpenStreetMap in QGIS version 3.24 [26].

Data from control and CHAMU calls did not show normal distributions, as assessed by the Kolmogorov-Smirnov test [27]; thus, comparisons were made using the Mann-Whitney test at 5% significance level (p≤0.05).

## 3 Results

### 3.1 Design thinking

**Fig 2:**
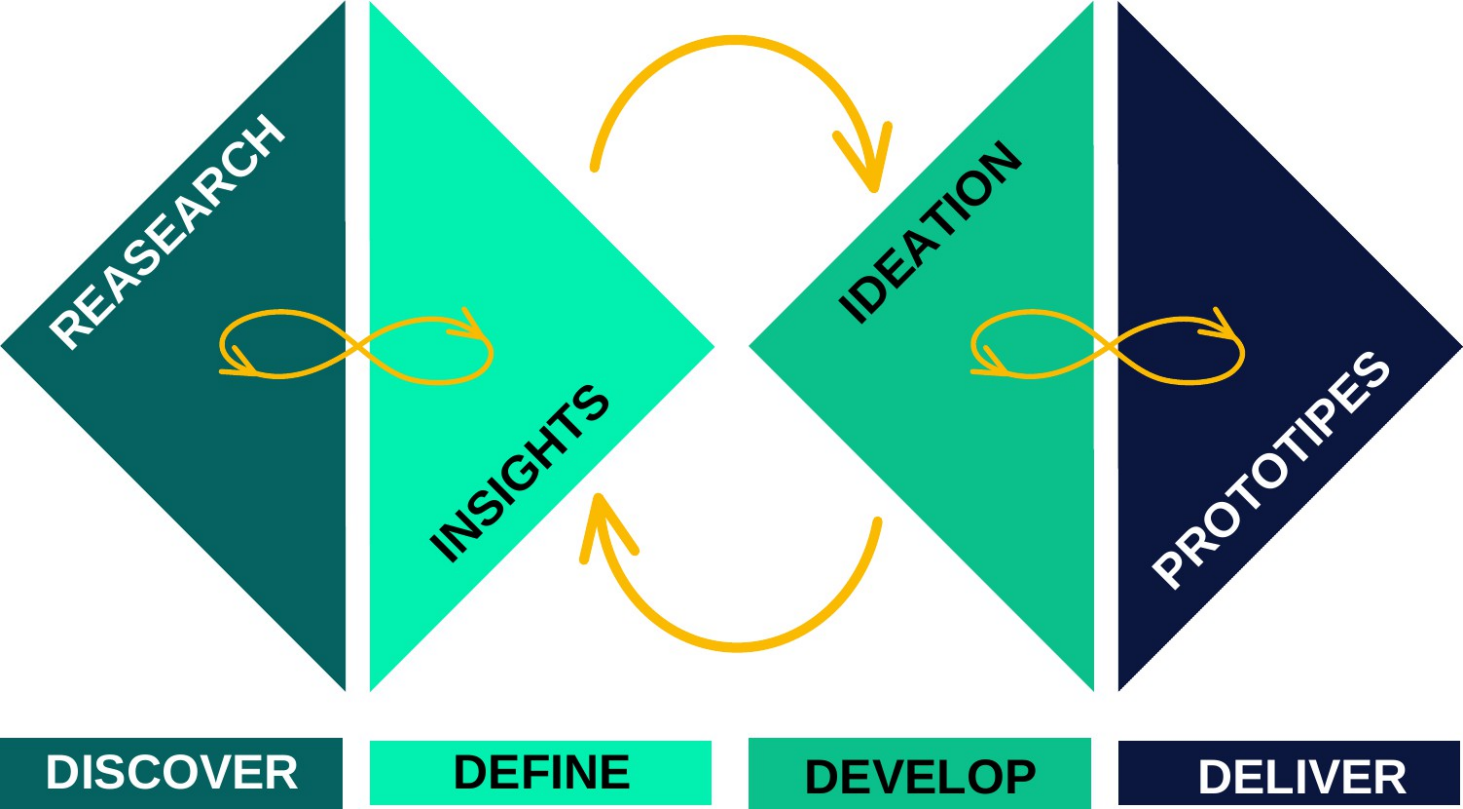
Double Diamond method (adapted from the British Design Council [6]).

Following the cyclical perspective of the design thinking method with the Double Diamond approach [6], as shown in Fig 2, we developed the “CHAMU192” app. The name of the application is derived from the combination of the Portuguese words “chamar” (call) and “SAMU” (EMS), associated with the number 192, the emergency number in Brazil (equivalent to 911).

#### 3.1.1 Data gathering: Discover and define

Descriptive analysis of the results of the EMS team interview revealed a major, difficulty in obtaining the location or complete address of emergency occurrence.

The number of individuals on general population who participated in the survey was 266. The mean age of the sample was 33.64 ± 14.80 years old, with a range of 18 to 73 years old. The survey showed that 200 participants (75.4%) would not know how to provide the address of their whereabouts in a given emergency situation without using geolocation tools.

#### 3.1.2 Development

**Fig 3:**
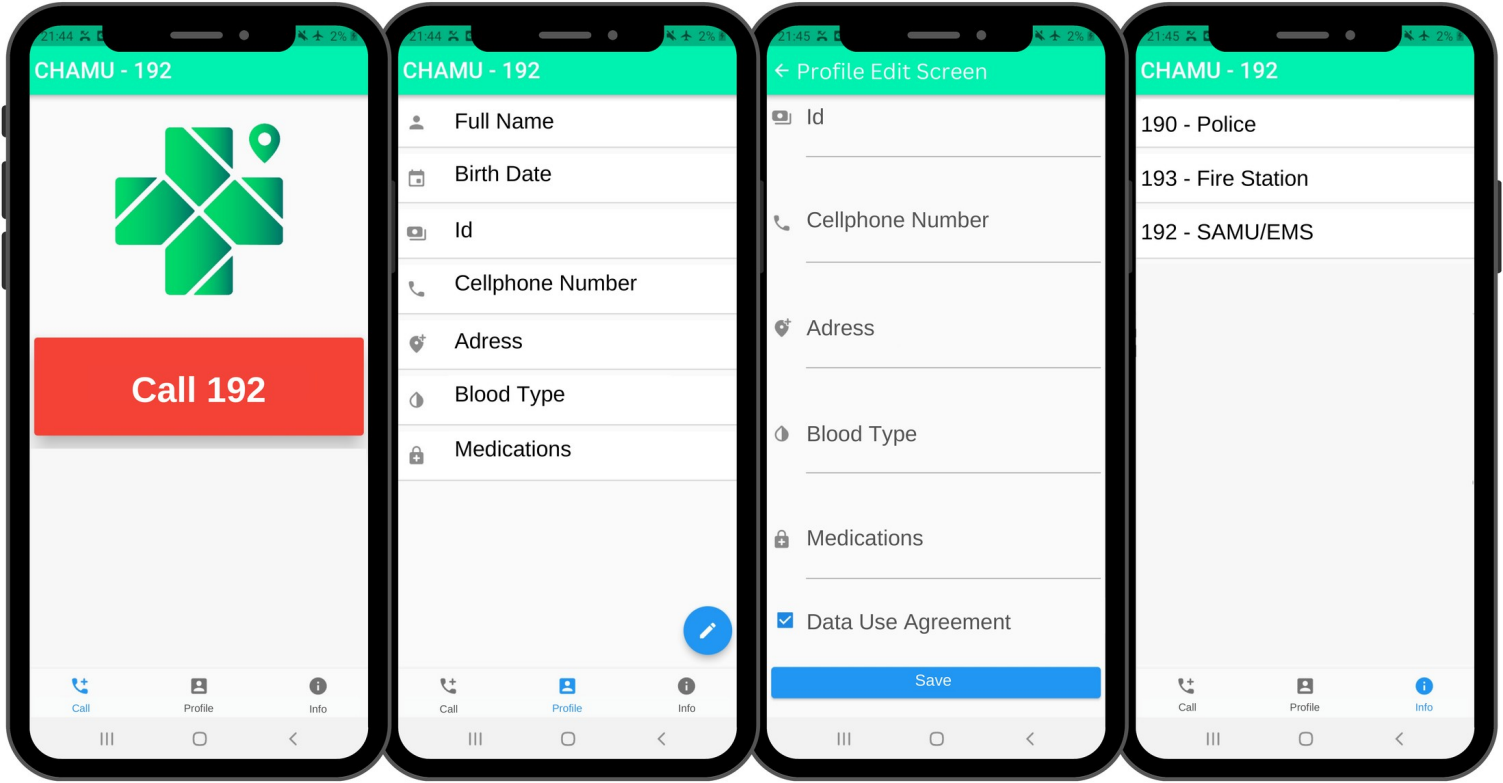
CHAMU192 screens, from left to right: Call screen, profile screen, profile editing screen and information screen.

The CHAMU192 user interface was created for the Android operating system (version 7.1.2 Nougat onward) using Flutter [15]. The app was designed to have the following screens: “Call’’, “Profile”, “Profile Edit” and “Information” (Fig 3). The “Call Screen” is the first screen the user sees when opening the app. It contains a button for making an emergency call. The “Profile Screen”, is the second screen, whereby users may enter their demographic information by clicking a button, redirecting them to the “Profile Edit Screen”. Here the user may add their registration information, which includes their full name, date of birth, ID number, telephone, address, blood type and medications in use, if any. There is a checkbox for the user to agree to the terms of data use for protection, confidentiality and secrecy. After the information is inserted and the checkbox marked, the user can press a button to save their information.

When registering, the user will need to accept the Data Use Agreement, as required by the General Data Protection Law [10] and allow their location and information to be shared while using the app. The last screen on the app is the “Information screen” which provides public service numbers, such as that of the civil police, municipal guard, and fire department, and other useful information for emergency situations.

When a call is made using the “CALL 911” button, the information contained in the CHAMU192 profile is sent to the MRATO screen, as well as the real-time geolocation of the device making the call. For this, the latitude and longitude coordinate system are used to plot the location on a map of the region. At the same time that coordinates are sent to call-takers, the user’s call is automatically redirected to the EMS conventional number, allowing them to speak directly to call-takers. Therefore, by the time users begin to speak with call-takers, the location of the user is already known, allowing callers to focus on the details of the event.

### 3.2 Evaluation of the CHAMU192 user interface by experts

The panel of experts was composed of four women and four men (n = 8). All evaluators were in the 30 to 50 years age group, and had postgraduate (37.5%), master’s (37.5%) and/or doctoral (25%) degrees. Half of experts had more than 10 years of experience in the Urgency and Emergency area, with 87.5% working in EMS.

**Fig 4:**
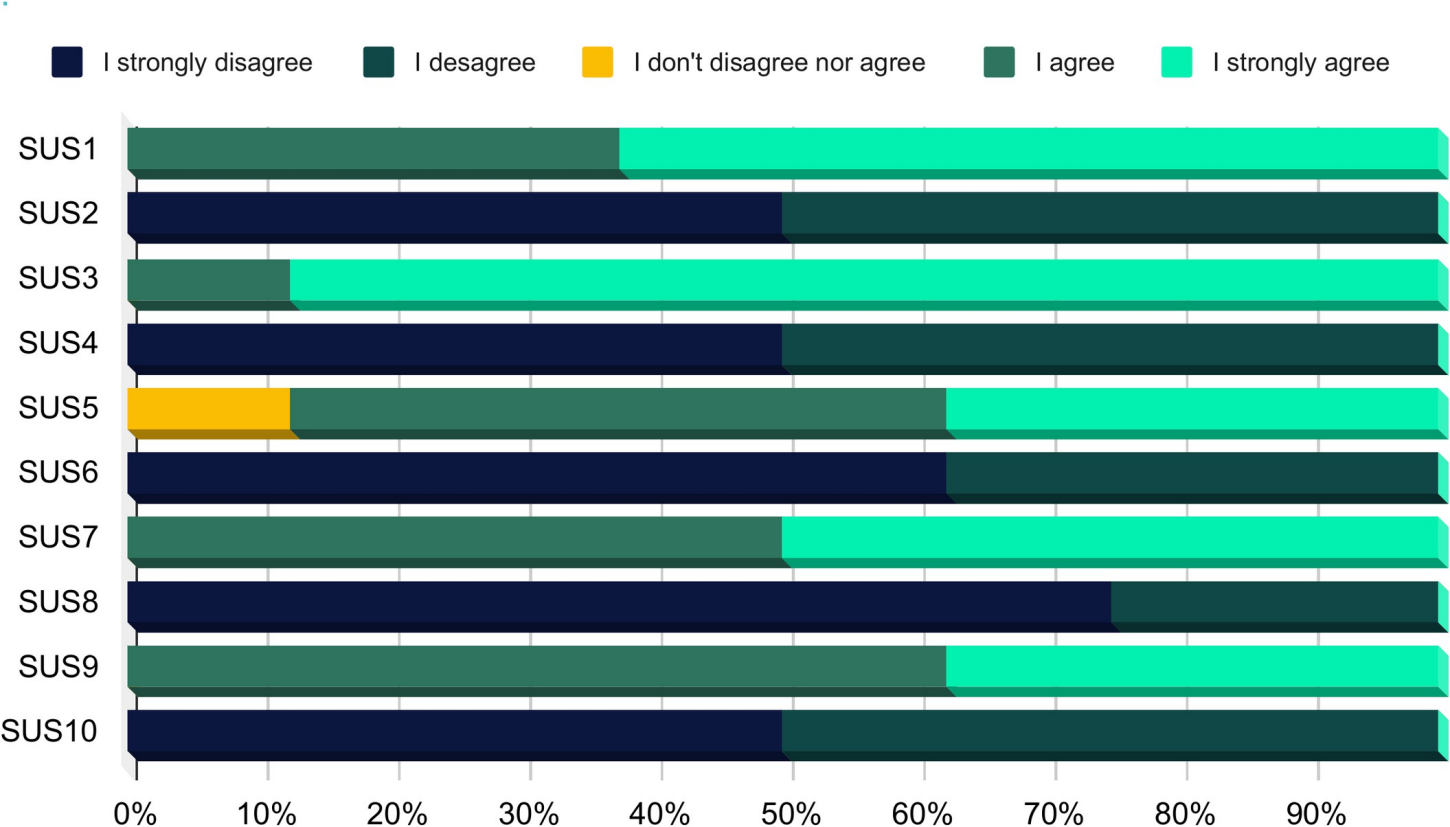
Expert evaluation of the proposed geolocation app, as assessed by the System Usability Scale.

The mean score of experts was 90, representing a “best imaginable score” in terms of app usability. CVC_t_ was 0.89 (the minimum for 8 specialists is 0.83). As shown in Fig 4, odd-numbered items, considered positive affirmations, were scored as agree or strongly agree by 97.5% of experts. Even numbered items, considered negative, were scored as disagree or strongly disagree by 100% of experts, indicating a positive evaluation of the app.

**Fig 5:**
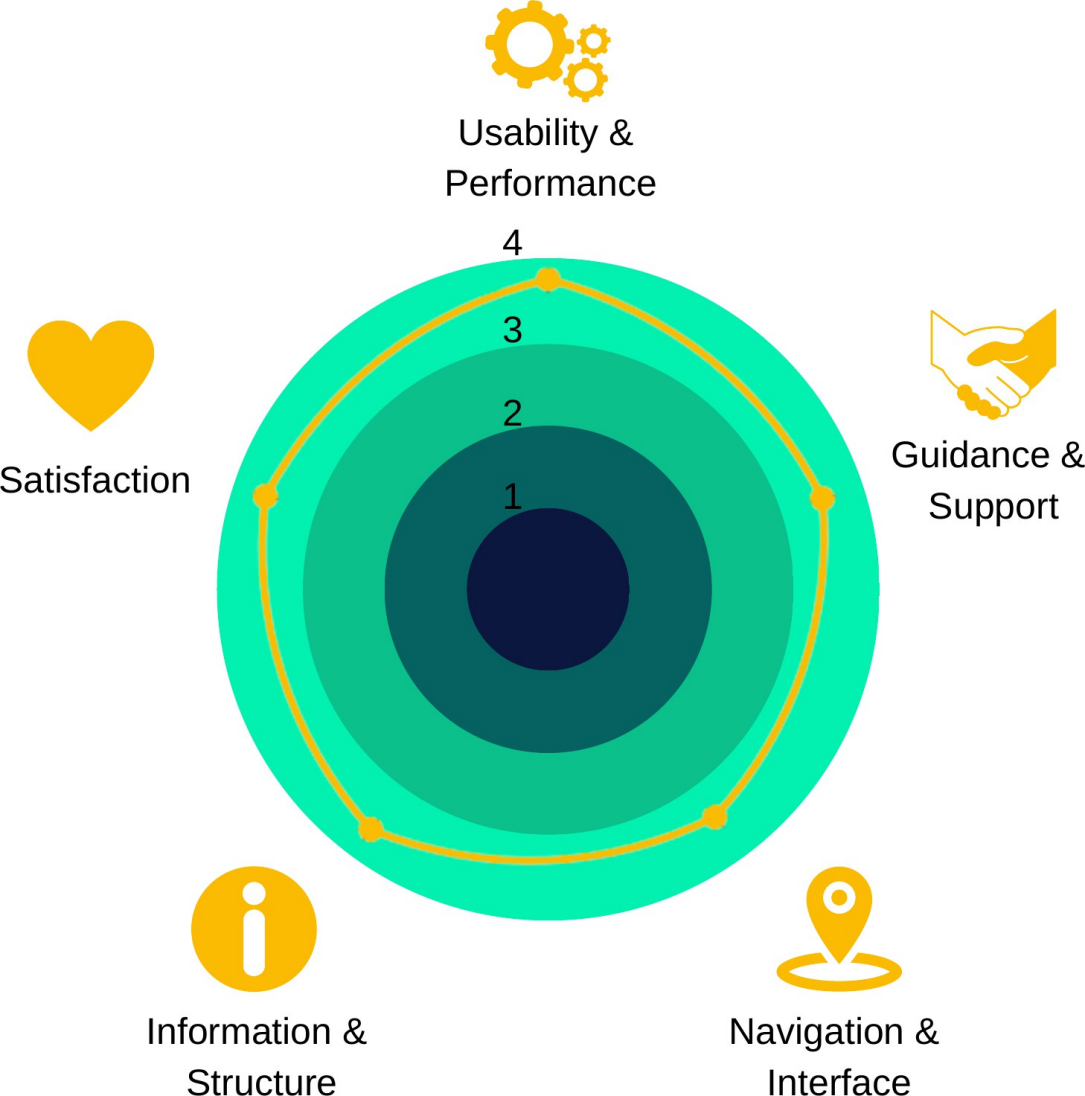
Radar chart for System Usability Scale results associated with e-health criteria.

For better visualization of SUS result, e-health criteria were adopted [28]. Items 1 and 3 were classified as “Usability and Performance”; 2 and 4 were as “Guidance and Support”; 5 and 6 as “Navigation and Interface”; 7 and 8 as “Information and Structure”; and 9 and 10 as “Satisfaction”. The radar chart (Fig 5) show that the app had a positive evaluation in all criteria.

### 3.3 Comparison of conventional call times and app-mediated call times

The median call time of the 2,326 calls classified as urgency and emergency was 222 s. Of these calls, 350 were randomly selected and analyzed as the control group. The T2d was determined for each call, with a median of 35.67 s (26.00–48.12s), and a maximum of 196.40 s.

**Fig 6:**
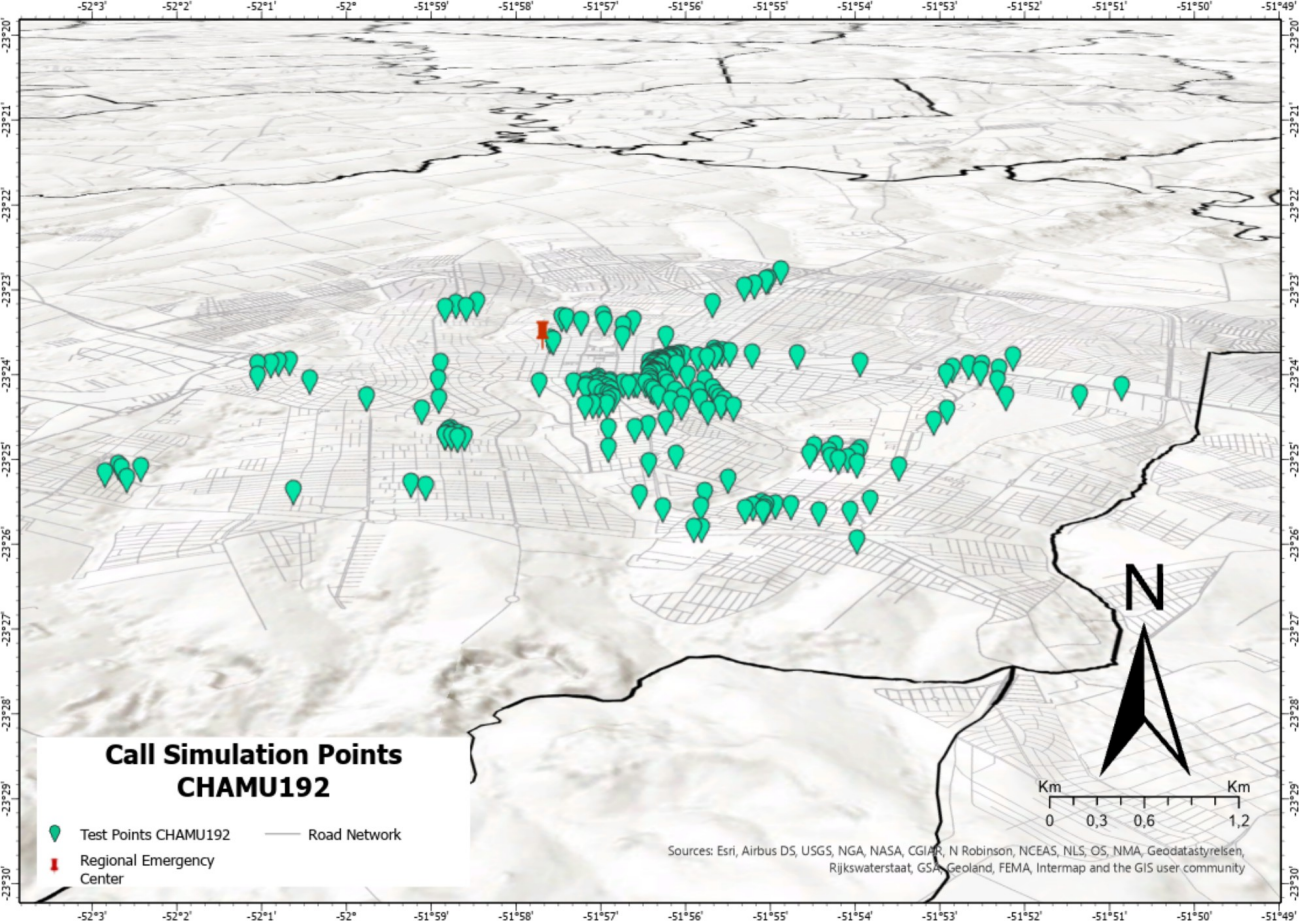
Geolocation of test calls made via CHAMU192, plotted using QGIS OpenStreet map.

The latency time for the user geolocation to reach MRATO via CHAMU192 was determined in 201 test calls conducted in Maringá, Paraná state, Brazil (Fig 6). The median time to send the geolocation data was 0.20 s (0.15–0.24 s), and the maximum 0.30 s.

**Fig 7:**
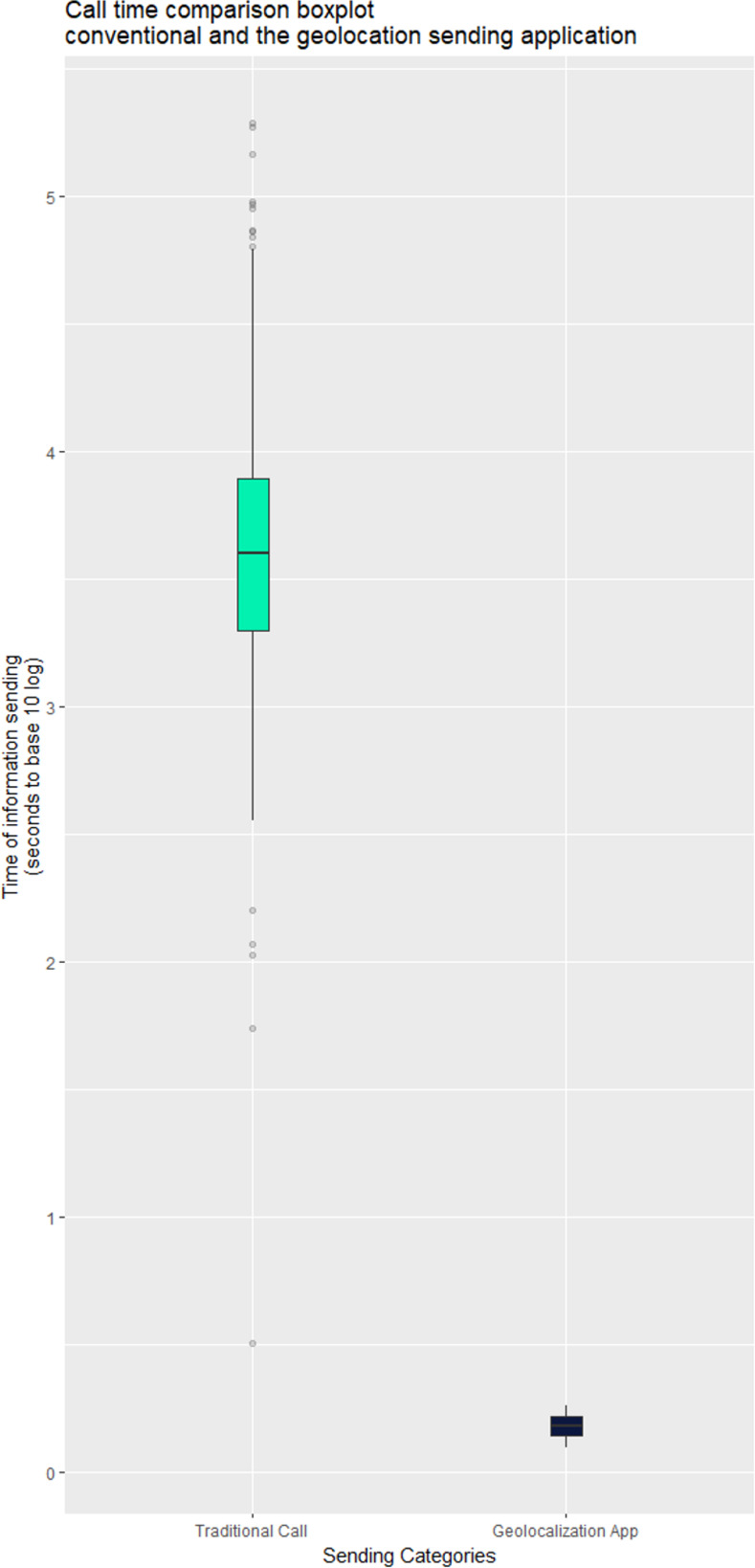
Boxplot showing median and quartiles of control and CHAMU192 call times.

CHAMU192 calls had significantly lower response time than control calls, as assessed by Wilcoxon Mann-Whitney test (0.20 s vs. 35.67 s, p<0.0001). A Boxplot chart of the results is shown in Fig 7, together with medians and quartiles.

## 4 Discussion

Incorporation of technologies in hospitals and health systems is a complex task for decision-makers, involving multiple factors, such as cost, patient safety, professional training, and social and cultural aspects. To the best of our knowledge, this is the first study to analyze and compare the time taken to relay the address or location of an emergency situation through conventional EMS calls with a validated geolocation mobile application based on GPS in a medium-large city in southern Brazil.

Currently there are some geolocation apps related to emergency services available on the market, such as PulsePoint [29] and Integrated Emergency Response [30]. However, each app has its particularities. For instance, PulsePoint is used for cardiac arrest calls, not covering other urgencies. Furthermore, the apps not available for use in Brazil, nor are their codes available for performance assessment. Such an app must be compatible with the Brazilian Unified Health System and SAMU databases for correct assessment of improvement in emergency call response times in Brazil. Therefore, here, we developed and validated CHAMU192.

Our results demonstrate that CHAMU192 significantly reduces the time taken by MRATO acquire location data, as the app provides the geolocation data almost instantaneously to the EMS, precluding the need for the caller to pass this information verbally to the call-taker (Td2). The differences in efficiencies between the conventional call method and the app-mediated call demonstrate that some standard protocols are maintained by tradition, even though there is no scientific evidence of the advantages of these methods.

Using the app, we observed a 99.44% decrease in T2d compared with conventional call. The Wilcoxon Mann-Whitney test confirmed that CHAMU192-mediated calls provide location data significantly faster than the conventional method.

Delays in PEC response times and subsequent delays in treatment can increase the risk of poor outcomes for time-dependent emergencies such as severe polytrauma, stroke and cardiopulmonary arrest (CPA) [31]. In CPA, each minute without defibrillation decreases the patient’s survival by up to 10%, which means that delays in conventional emergency calls due to difficulties in collecting geolocation data contributes to the increase in morbidity and mortality among victims of out-of-hospital CPA [32, 33].

Of the calls analyzed in our study, 74.11% were longer than the recommended (3 min or 180 s), with a mean of 222 s. We also found that the major part of the T2 time is attributed to T2d (59.45%), which is the time it takes for call-takers to obtain the caller’s address. A reduction in Td2 may translate into fewer sequelae and complications in victims of car accidents, a decrease in the length of hospital stays, and a consequent reduction in the total cost of treatment [34]. Therefore, any measure to minimize the response time must be taken in order to reduce morbidity and mortality [35].

During an emergency call, the caller often has difficulty expressing the location of the emergency accurately [36]. These findings probably explain the delays found in Td2 observed here. One of the limitations of the proposed method is that, individuals without access to a smartphone connected to internet, or those who have difficulty handling this type of technology, must continue using the conventional method of requesting EMS, through the tool-free call to 192. Given this limitation, the proposed too intended to complement, not replace, conventional telephone calls to the EMS.

There were also some limitations associated with the use of retrospective secondary data, such as low number of calls analyzed and sampling of calls from a single emergency system. These factors may reflect a specific scenario, hindering the use of the app developed here in other situations. Although CHAMU192 may improve emergency care, especially in low- and middle-income countries, it is necessary to further evaluate its applicability using datasets from other services.

Some future perspectives include the development of partnerships with public and private health networks on municipal, state and federal scales. Tools that can measure and reduce the response time of the potential to help decision-makers in the health sector assess the impact of incorporating new frugal technology and fulfill requirements to invest in research and experiences with new technologies. Investigation of the effectiveness of the app by using comparative analysis, testing, improvements and version updates will remain a priority of the research group.

## 5 Conclusion

Emergency calls made through CHAMU192 resulted in significant shorter service time compared with conventional calls. The use of the app could potentially reduce PEC response times. The finding presented here may serve as a basis for e-health apps to be inserted in society with scientific support, seeking real improvements that can be perceived by the population and by public private health managers.

## Supporting information

S1 Appendix The System Usability Scale (SUS). (DOCX)

S1 Checklist Additional information regarding the ethical, cultural, and scientific considerations specific to inclusivity in global research. (PDF)
