# Supplementary material for: Designing, development and validation of an app to reduce the response time of the emergency medical services
Source: PLoS One. 2024 Mar 25;19(3):e0299828. doi: 10.1371/journal.pone.0299828 (PMC10962839; doi:10.1371/journal.pone.0299828)
Supplement: S1 Appendix — (DOCX) [file pone.0299828.s001.docx]

**SYSTEM USABILITY SCALE**

1.I think that I would like to use this system frequently.:

| **I strongly disagree** | **I disagree** | **neutral** | **agree** | **completely agree** |
| --- | --- | --- | --- | --- |
| 1 | 2 | 3 | 4 | 5 |

2.I found the system unnecessarily complex:

| **I strongly disagree** | **I disagree** | **neutral** | **agree** | **completely agree** |
| --- | --- | --- | --- | --- |
| 1 | 2 | 3 | 4 | 5 |

3. I thought the system was easy to use:

| **I strongly disagree** | **I disagree** | **neutral** | **agree** | **completely agree** |
| --- | --- | --- | --- | --- |
| 1 | 2 | 3 | 4 | 5 |

4. I think that I would need the support of a technical person to be able to use this system:

| **I strongly disagree** | **I disagree** | **neutral** | **agree** | **completely agree** |
| --- | --- | --- | --- | --- |
| 1 | 2 | 3 | 4 | 5 |

5.I found the various functions in this system were well integrated:

| **I strongly disagree** | **I disagree** | **neutral** | **agree** | **completely agree** |
| --- | --- | --- | --- | --- |
| 1 | 2 | 3 | 4 | 5 |

6. I found a lot of inconsistency in this app:

| **I strongly disagree** | **I disagree** | **neutral** | **agree** | **completely agree** |
| --- | --- | --- | --- | --- |
| 1 | 2 | 3 | 4 | 5 |

7. I would imagine that most people would learn to use this system very quickly:

| **I strongly disagree** | **I disagree** | **neutral** | **agree** | **completely agree** |
| --- | --- | --- | --- | --- |
| 1 | 2 | 3 | 4 | 5 |

8. I found the system very cumbersome to use:

| **I strongly disagree** | **I disagree** | **neutral** | **agree** | **completely agree** |
| --- | --- | --- | --- | --- |
| 1 | 2 | 3 | 4 | 5 |

9. I felt very confident using the system:

| **I strongly disagree** | **I disagree** | **neutral** | **agree** | **completely agree** |
| --- | --- | --- | --- | --- |
| 1 | 2 | 3 | 4 | 5 |

10. I needed to learn a lot of things before I could get going with this system:

| **I strongly disagree** | **I disagree** | **neutral** | **agree** | **completely agree** |
| --- | --- | --- | --- | --- |
| 1 | 2 | 3 | 4 | 5 |
